# Ganoderic Acid A Alleviates OVA-Induced Asthma in Mice

**DOI:** 10.1007/s10753-021-01468-1

**Published:** 2021-05-26

**Authors:** Xinhua Lu, Chenyang Xu, Rui Yang, Guojun Zhang

**Affiliations:** 1 Respiratory and Critical Care Medicine, The First Affiliated Hospital of Zhengzhou University, Zhengzhou, 450052 China; 2 Henan Luoyang Orthopedic-Traumatological Hospital, Luoyang, 471000 China

**Keywords:** ganoderic acid A, asthma, inflammation, TLR/NF-kB

## Abstract

The aim of this study is to investigate the effects of ganoderic acid A (GAA) on OVA-induced asthma in mice. Mouse asthma model was established by ovalbumin (OVA) in vitro. Diff-Quik staining was used to observe the total numbers of cells and the number of classification cells in each group, and HE staining was used to observe lung inflammation in lung tissue sections. ELISA was used to detect the effect of GAA on the levels of interleukin-4 (IL-4), IL-5, and IL-13 in serum and lung tissue. The expression levels of TLR/NF-κB were detected by Western blot. Immunohistochemistry was used to observe the expression changes of TLR4 and P-P65. Compared with the normal group, the inflammatory cell count, IL-4, IL-5, and IL-13 expression in the model group increased, and TLR/NF-kB signal protein expression increased. Compared with the model group, in GAA group, the number of inflammatory cells, the expression of IL-4, IL-5, and IL-13 decreased, and the expression of TLR/NF-kB signaling protein decreased. GAA regulated lung inflammation in asthmatic mice by inhibiting TLR/NF-kB signaling pathway.

## INTRODUCTION

Bronchial asthma is a chronic disease characterized by pathological changes such as abnormal inflammatory reaction of the airway, abnormal changes in airway wall structure, and increased tracheal-bronchial reactivity [1]. In recent years, according to the World Health Organization (WHO) survey, there are about 15 million people who suffer from acute attacks of bronchial asthma, affecting their quality of life and losing their ability to work every year, while about 250,000 patients worldwide die of asthma every year [2]. In China, there are at least 30 million patients with asthma. Most patients begin to suffer from asthma before the age of 5. The proportion of children suffering from asthma before the age of 3 accounts for about 50%, especially in regions or seasons with severe fog, which poses a serious threat to asthmatic patients, and the incidence rate of asthma tends to increase by 3–5%. Therefore, further discussion on the inflammatory characteristics of asthma and early prevention and control measures are currently the focus of research and attention of respiratory disease experts all over the world [3].

Asthma is the result of the interaction between endogenous immune system and exogenous immune system [4]. Allergens in the environment, such as indoor dust storm (HDM), endotoxin, and virus infection, which can be combined with pathogen-related pattern recognition receptors of the bodies, and different gene expression patterns are induced by activating different signal transduction pathways, thus causing the release of various proinflammatory factors [5]. On the one hand, various antigen-presenting cells (APC) in the body are stimulated to activate the innate immune response in the body; on the other hand, these inflammatory mediators participate in the regulation of T lymphocyte differentiation, which promote the occurrence and development of specific antigen-induced acquired immune response, and aggravate the inflammatory response of the body [6]. Human Toll-like protein was first discovered by Medzhitov et al. in 1997. Up to now, many studies have found that at least TLR_1–10_ exists in Toll receptor families. Different TLRs make different genes express after being stimulated, playing an important role in identifying self- and foreign antigens and harmful and harmless antigens [7]. The discovery and research provided new ideas for the treatment of a variety of immune function-related disorders such as bronchial asthma and allergic bronchopulmonary aspergillosis (ABPA) and enable people to have further understanding and understanding of the interaction between the two immune systems [8]. House dust female (HDM) is one of the major ligands of TLR4, which is ubiquitous in the air and induce acute attack of asthma. After TLR4 recognizes with HDM in the environment, it can activate intracellular transcription factor NF-kB and related protein kinases; release related cytokines, such as TNF-a, IL-8, and IL-6; and promote the metamorphosis of the body [8]. At the same time, activated APC, such as alveolar giant cells in the airway, can further affect the transcription activity of inducible NOS and promote the production of NO, which activates the intracellular bactericidal mechanism in the body and further aggravates tissue damage. Therefore, APC has set up a bridge for the body’s immune response [9]. *Ganoderma lucidum* is a valuable traditional Chinese medicine. *Ganoderma lucidum* (scientific name, *Ganoderma lucidum* Karst), also known as Linzhongling and Qiongzhen, is the fruiting body of *Ganoderma lucidum*, a fungus of the Polyporaceae [10]. It has effects in invigorating qi, tranquilizing mind, relieving cough and asthma, and prolonging life. It can be used for treating vertigo, insomnia, palpitation, short breath, neurasthenia, asthenia, cough, and asthma [11]. Ganoderic acid A (GAA) is the main component of *Ganoderma lucidum*, which shows a variety of pharmacological activities and has the effects of reducing blood lipid, lowering blood pressure, protecting liver, regulating liver function, etc. [12]. However, there are few reports of GAA on OVA-induced asthma. The purpose of this paper is to explore the effect of GAA on OVA-induced asthma in mice and its mechanism.

## MATERIALS AND METHODS

### Reagents

OVA, PMSF, and PIRA lysates were purchased from Sigma Company in the USA. IL-13 ELISA test kits for IL-4, IL-5, and IL-13 were purchased from Invitrogen Company in the USA. Therefore, all antibodies were purchased from Cell Signaling Company in the USA.

### Animals

Fifty female BALB/c mice (18–22 g) were provided by the Laboratory Animal Center of Zhengzhou University. They were reared for 1 week under the conditions of constant temperature (23–25°C), relative humidity (40–60%), and 12-h light/dark cycle. This study was carried out in accordance with the recommendations of Zhengzhou University. The protocol was approved by the Zhengzhou University.

### Establishment and Grouping of Asthma Models

Mice were randomly divided into control group, model group, dexamethasone (Dex, 2 mg/kg ), and GAA (20, 40 mg/kg). According to the method of Lee et al. [13] and modified to prepare asthma model, normal mice were injected intraperitoneally with a mixture of 10-mg OVA + 1-mg aluminum hydroxide and 200 μl of normal saline on the 1st, 7th, and 14th days. On the 21st day, sensitized mice were placed in a glass cover, and each group was atomized and excited with 0.1-g OVA + 10-ml normal saline for 30 min once/d for a total of 7 days. HLOL treatment group mice were treated with the same model group mice, and 20, 40 mg/kg of GAA was administered 1 h before each challenge of immunity. Control mice were injected intraperitoneally with the same dose of normal saline instead of OVA and stimulated immunity.

### Detection of Airway Hyperreactivity (AHR )

After 24 h of the last OVA booster immunization, the sober mice were placed in a barometric volume recording room, and the average baseline reading within 3 min was recorded. Acetylcholine was used for atomization for 3 min, and the average reading was recorded. Enhanced pause (Penh) was calculated according to the manufacturer’s plan as airway constriction index to reflect the degree of increased airway reactivity.

### Determination of IgE

The serum stored at −80 °C were taken out, thawed on ice, centrifuged at 4 °C for 10 min, and the IgE level in serum was detected by ELISA method, strictly according to the instruction of ELISA kit.

### Determination of IL-4, IL-5, and IL-13 in Serum

According to the ELISA kit instruction steps, calculate the content of each group according to the standard curve.

### Pathological Examination and Pathological Image Analysis of Lung Tissue

Lung tissue sections were prepared, stained with HE according to the kit instructions, and observed under ordinary optical microscope; the score of inflammatory cell infiltration around bronchus was made, and the judging criteria were (1) no inflammatory cell (0 score); (2) a few inflammatory cells (1 point); (3) more inflammatory cells with uneven distribution (2 points); (4) a large number of inflammatory cells are evenly distributed and rarely aggregated into clusters (3 points); and (5) a large number of inflammatory cells clustered (4 points). Two pathologists read the films blindly, and each group measured ≥ 25 airways.

### Immunohistochemical

Paraffin sections are routinely dewaxed and hydrated, and 0.3% H_2_O_2_ methanol treatment inactivates endogenous peroxidase. According to the antibody specification, rabbit anti-mouse TLR4 and p-NF-kBp65 antibodies (1:1000) were prepared proportionally, incubated overnight at 4 °C, washed with PBS, and incubated with biotin-labeled goat rabbit IgG as secondary antibody for 30 min, DAB developed color, hematoxylin counterstained after washing with running water, washed back to blue with running water, observed under a mirror after sealing, and photographed. At high magnification, IHC staining showed brown–yellow staining as positive cells, and image J was used for statistical analysis.

### Western Blot

The lung tissue was collected and lysed to obtain protein. The concentration of protein was determined by BCA method. Each lane was loaded with 20 μg of the total protein. The protein was separated on 12% SDS-PAGE gel for 120V and 90 min. The separated protein was transferred to PVDF membrane electrically at 250 mA and 90 min. The 50-g/L skim milk powder TBS-T buffer was incubated for 1 h for blocking. Primary antibodies were added respectively and overnight at 4 °C. On the second day, after washing the membrane, goat rabbit IgG labeled with avidin was hybridized and incubated at 37 °C for 1 H. After washing the membrane, ECL luminescent reagent was added, and Gel Doc software was used for image acquisition and data analysis.

### Statistical Analysis

The experimental results are expressed as mean ± standard deviation. The experimental data were statistically analyzed by SPSS 14.0 and tested by one-way ANOVA, *P* < 0.05.

## RESULTS

### GAA Attenuated Cellular Changes in Serum of Asthmatic Mice

**Fig. 1:**
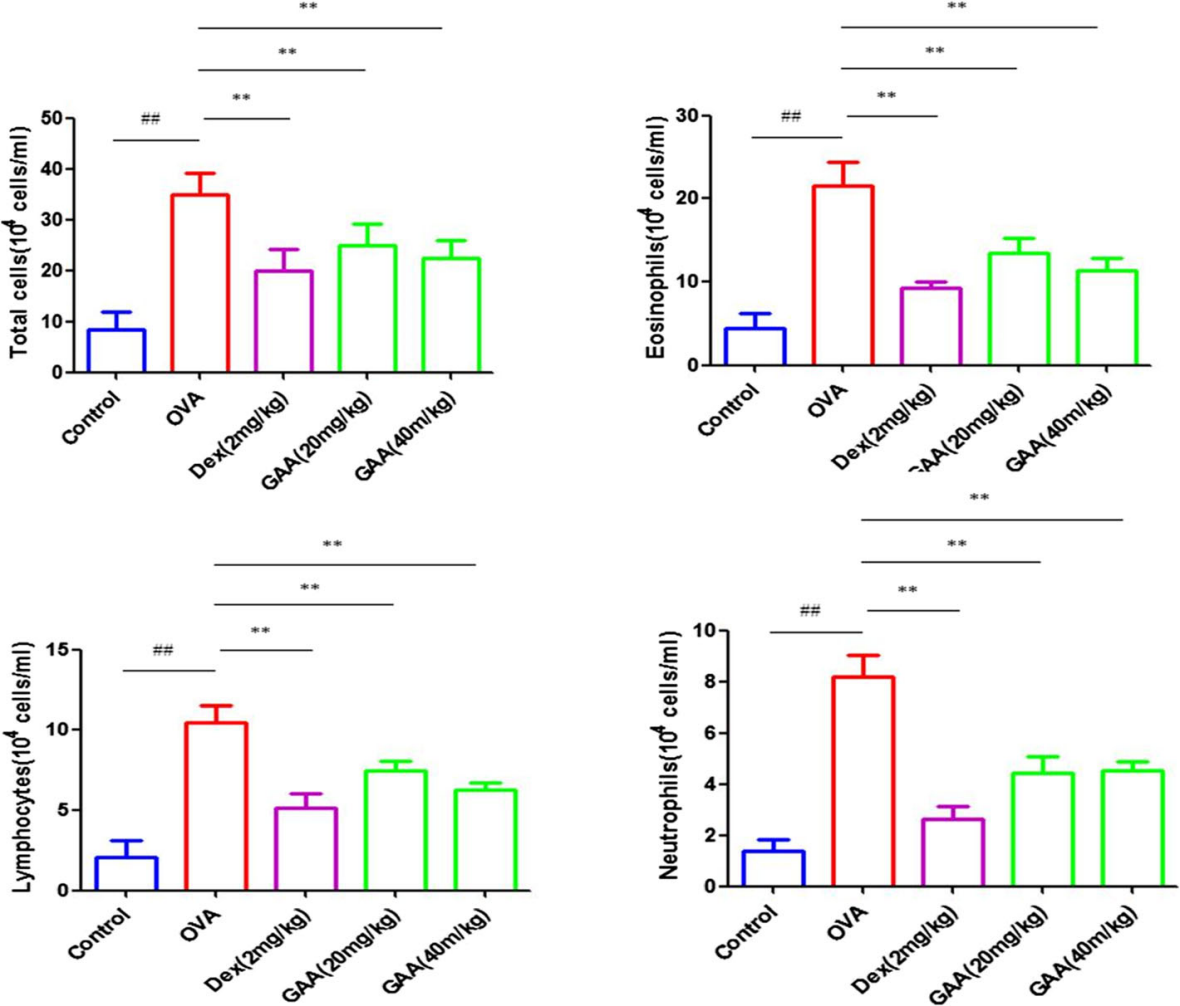
GAA attenuated cellular changes in serum of asthmatic mice. Values are expressed as means ± SEM. Compared with control: ^#^*P* < 0.05, ^##^*P* < 0.01; compared with OVA:*P < 0.05, **P < 0.01.

As shown in Fig. 1, compared with the control group, the total numbers of inflammatory cells, eosinophils, lymphocytes, and neutrophils in serum of the model group mice are significantly increased. However, the index in the GAA group was significantly decreased.

### Pathological Changes

**Fig. 2:**
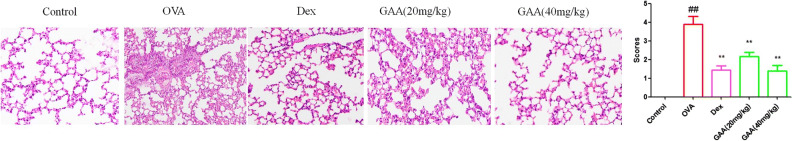
Pathological changes. Values are expressed as means ± SEM. Compared with control: ^#^*P* < 0.05, ^##^*P* < 0.01; compared with OVA:*P < 0.05, **P < 0.01.

HE staining showed that airway smooth muscle thickening was observed in lung tissue sections of model group mice, and a large number of inflammatory cell infiltration were observed around the bronchus and blood vessels. Compared with the control group, the inflammatory cell infiltration in lung tissue sections of asthmatic mice after intervention with GAA was significantly reduced, and the airway structure was relatively complete, indicating that anthocyanin alleviated OVA-induced airway inflammation (Fig. 2).

### IL-4, IL-5, and IL-13 Levels in Serum of Asthmatic Mice

**Fig. 3:**
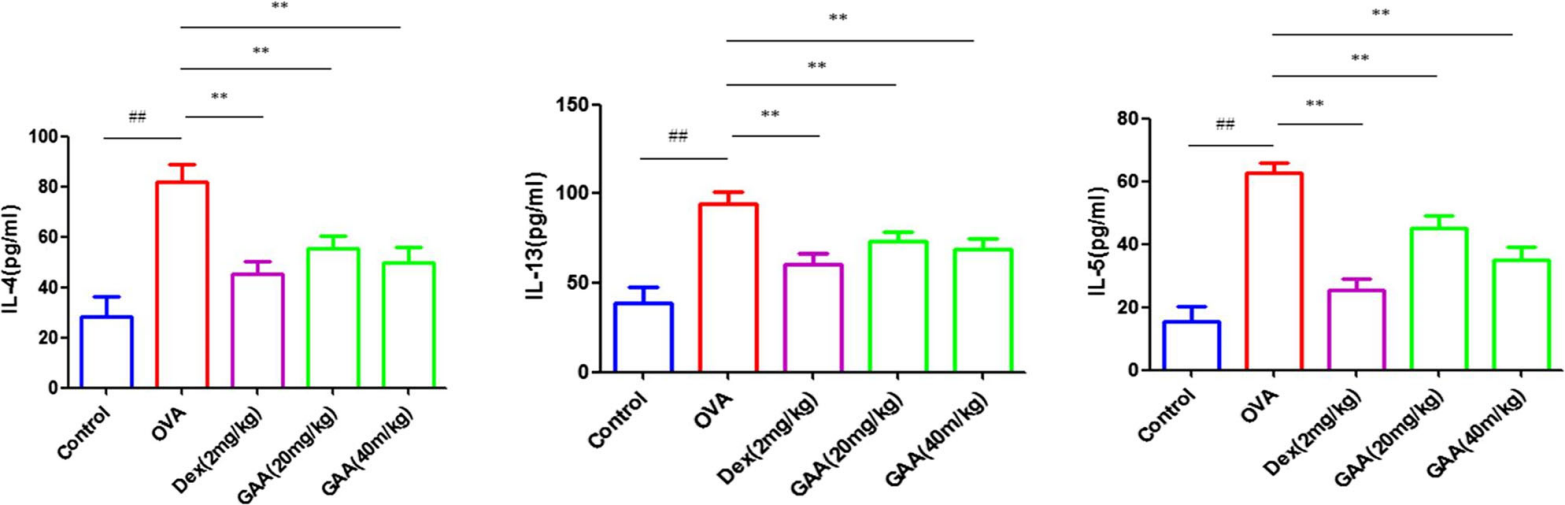
IL-4, IL-5, and IL-13 levels in serum of asthmatic mice. Values are expressed as means ± SEM. Compared with control: ^#^*P* < 0.05, ^##^*P* < 0.01; compared with OVA:*P < 0.05, **P < 0.01.

ELISA results showed that the levels of IL-4, IL-5, and IL-13 in the serum of model group mice were significantly higher than those of control group. The levels of IL-4, IL-5, and IL-13 in GAA group were lower than those in model group, as shown in Fig. 3.

### AHR in Asthmatic Mice

**Fig. 4:**
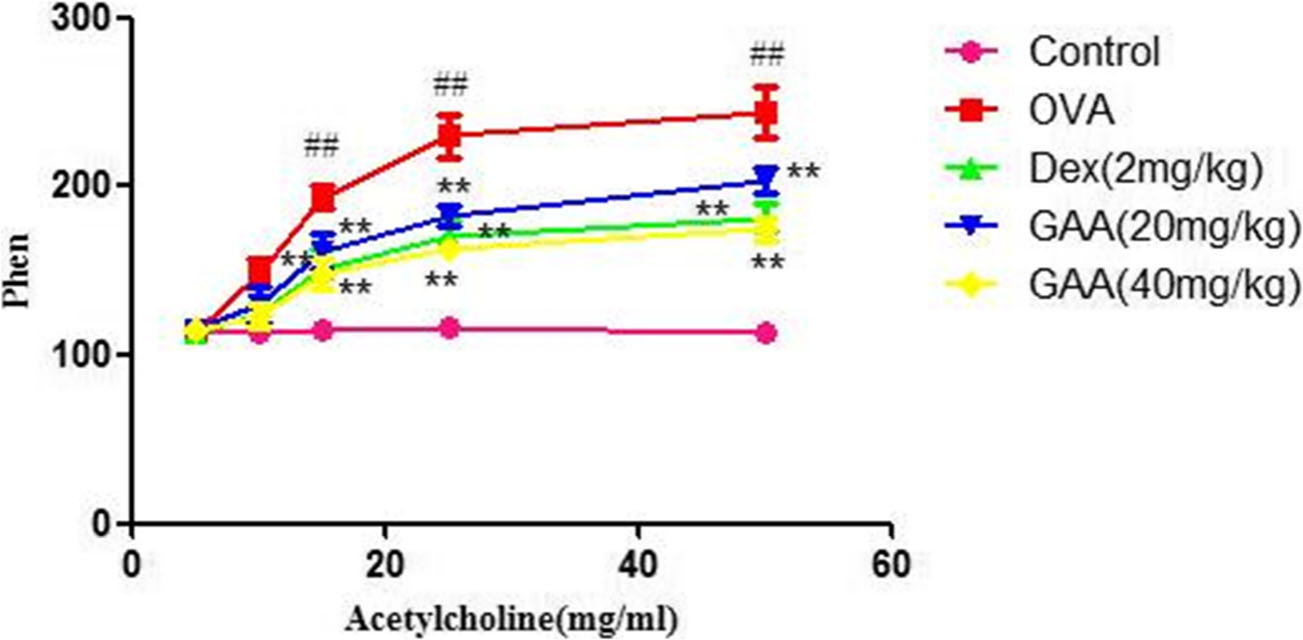
AHR in asthmatic mice. Values are expressed as means ± SEM. Compared with control: ^#^*P* < 0.05, ^##^*P* < 0.01; compared with OVA:*P < 0.05, **P < 0.01.

As shown in Fig. 4, compared with the model group, the Penh of the model group mice were higher than that of the control group. However, GAA significantly reduced Penh.

### Effect of GAA on IgE in Serum

**Fig. 5:**
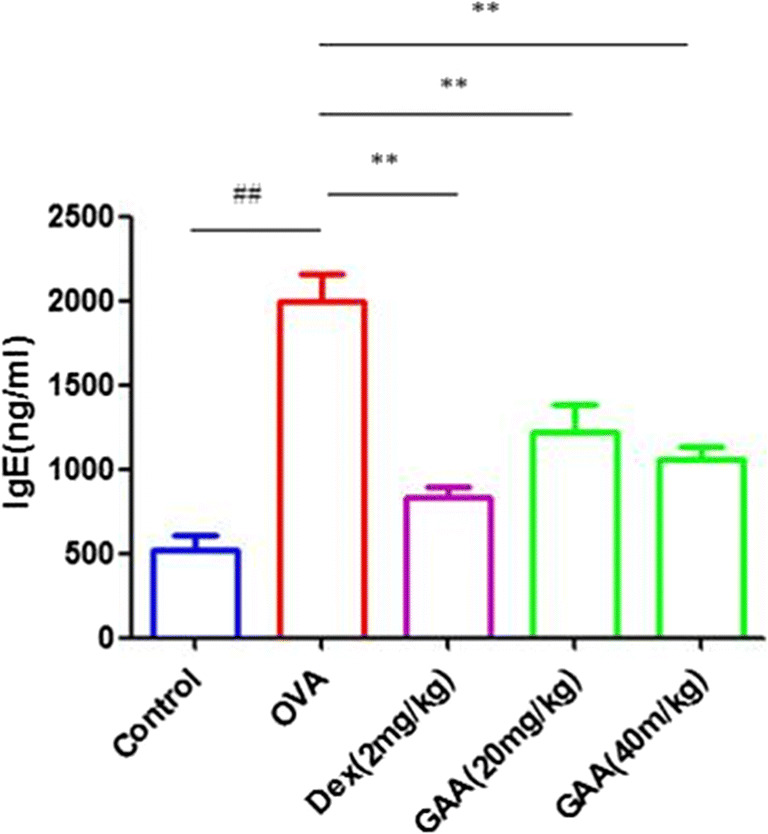
Effect of GAA on IgE in serum. Values are expressed as means ± SEM. Compared with control: ^#^*P* < 0.05, ^##^*P* < 0.01; compared with OVA:*P < 0.05, **P < 0.01.

Compared with the control group, IgE levels in serum in OVA-sensitized group were significantly higher than control group. Compared with OVA-sensitized group, the level of IgE in serum significantly decreased (Fig. 5).

### Effect of GAA on TLR/NF-kB Signaling Pathway in the Lung

**Fig. 6:**
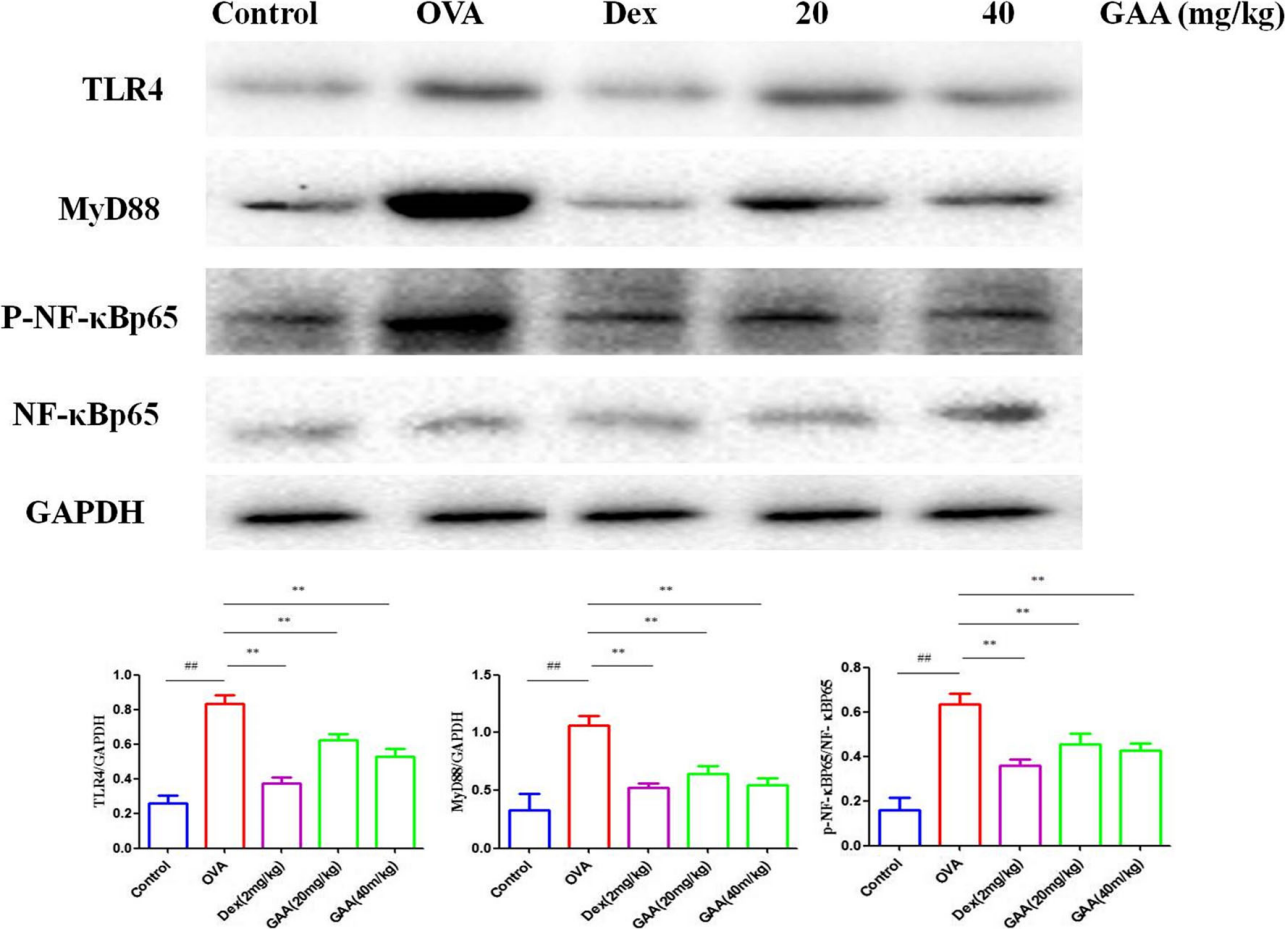
Effect of GAA on TLR/NF-kB signaling pathway in the lung. Values are expressed as means ± SEM. Compared with control: ^#^*P* < 0.05, ^##^*P* < 0.01; compared with OVA:*P < 0.05, **P < 0.01.

Compared with the control group, the levels of TLR4, MyD88, p-NF-kB, and p-IkBa in the lung in OVA-sensitized group significantly increased. Compared with OVA-sensitized group, the levels of TLR4, MyD88, and p-NF-kB significantly decreased in GAA group (Fig. 6).

### Effect of GAA on TLR4 and p-NF-kBp65 in the Lung

**Fig. 7:**
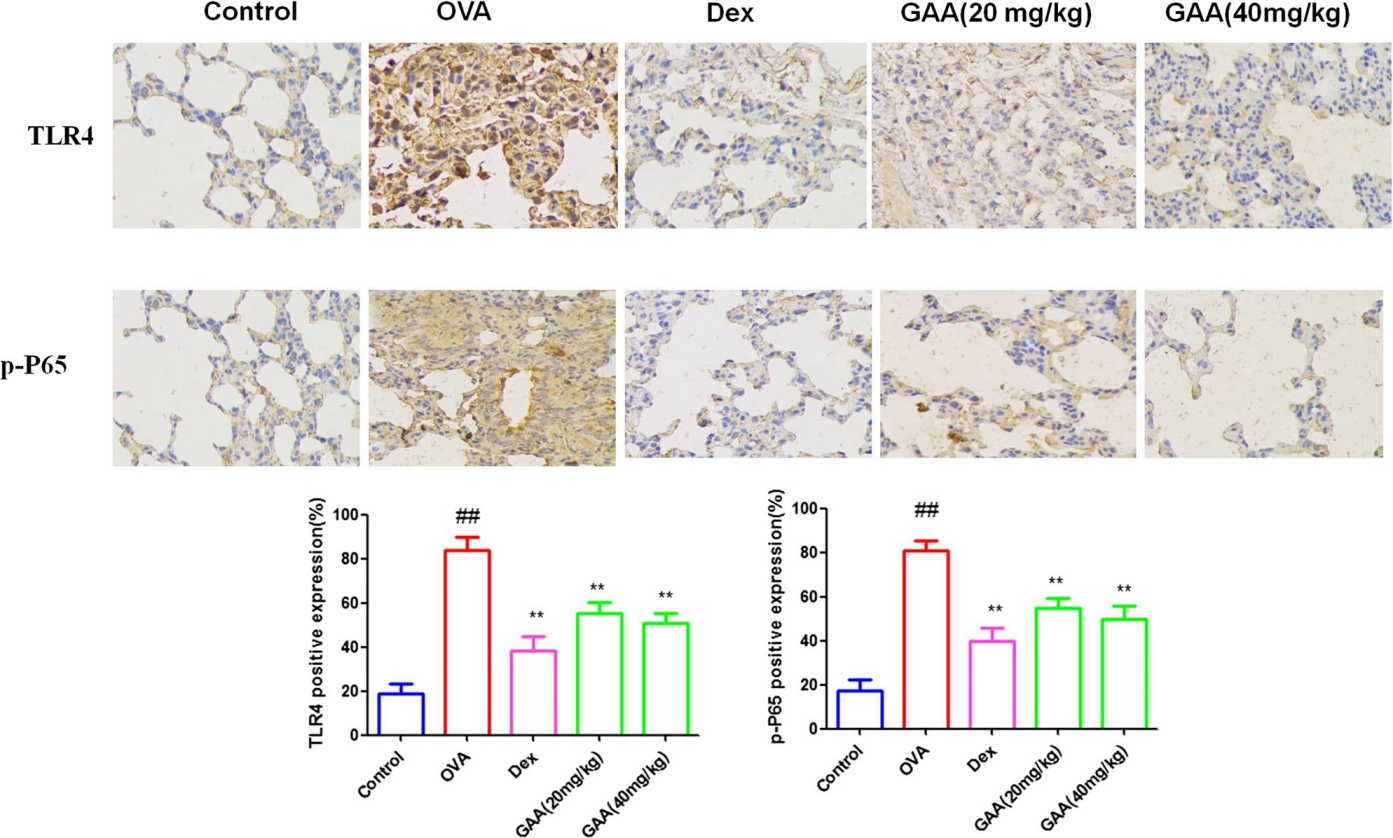
Effect of GAA on TLR4 and p-NF-kBp65 in the lung. Values are expressed as means ± SEM. Compared with control: ^#^*P* < 0.05, ^##^*P* < 0.01; compared with OVA:*P < 0.05, **P < 0.01.

Compared with the control group, the levels of TLR4 and p-NF-kB in the lung in OVA-sensitized group significantly increased. Compared with OVA-sensitized group, the levels of TLR4 and p-NF-kB significantly decreased in GAA group (Fig. 7).

## DISCUSSION

Asthma is an allergic airway inflammatory disease involving a variety of inflammatory mediators and cytokines. It is mainly characterized by Th1/Th2 and oxidation/oxidation imbalance, serum IgE, and eosinophilia and which can induce airway hyperresponsiveness and mucus hypersecretion and other symptoms [13, 14]. Asthma is a respiratory disease accompanied by lung injury. It is characterized by airway inflammation and bronchial hyperresponsiveness, which can eventually lead to lung failure due to airway remodeling [15]. In view of its inflammatory mechanism, glucocorticoid is currently the main recommended prevention and treatment method for asthma. The main effect of glucocorticoid on asthma is its strong anti-inflammatory effect [16]. However, its side effects such as secondary infection and osteoporosis should not be overlooked. However, with the long-term use of glucocorticoids, the more likely it is to progress into refractory asthma and hormone-dependent asthma. Therefore, it is urgent to need a drug that can treat asthma and effectively control airway inflammation of asthma, especially for hormone-resistant asthma. In our experiment, the mouse asthma model was successfully replicated, and GAA was used to intervene and detect TLR4/NF-κB-related pathway.

This study found that GAA inhibited the increase of inflammatory cells in BALF of asthmatic mice; reduced the levels of IL-4, IL-5, and IL-13 in serum and BALF; reduced the infiltration of inflammatory cells in lung tissue of asthmatic mice; and reduced airway hyperreactivity. These results show that GAA has obvious anti-asthma effects. On this basis, we further detected the levels of TLR/NF-κB, and the experimental results show that GAA significantly inhibited the activation of TLR/NF-κ B.

For asthma in infants and children, an important high-risk factor for its aggravation is the common virus invasion of various respiratory tracts, which aggravates inflammation [17]. The respiratory syncytial virus (RSV) is the most infectious disease. RSV aggravates acute asthma attacks in children in many ways, including recognition of RSV, CD14, and TlR4 complexes; activation of TLR4-induced signaling pathways; promotion of the body’s innate immune response; and enhancement of expression of various inflammatory mediators, chemokines, and adhesion molecules. Therefore, the integrity of TLR4 molecular structure and the expression of signal function are the key links to ensure smooth intracellular transmission of external stimuli. The experimental results show that GAA significantly decreased the levels of TLR4.

TLR4, as a natural immune recognition receptor, recognizes the signal cascade caused by the receptor, which leads to the activation of NF-κB-dependent pathways, causing the expression of many inflammatory factor genes. These expressed inflammatory factors reactivated NF-κB expression, forming snowballing positive feedback regulation [18]. The expression of NF-κB is widely found in various cells. Normally NF-κB is inactivated due to its binding with its inhibitor IkB. However, in abnormal state, when the epithelium is stimulated and damaged (such as allergic diseases), IkB is separated from NF-κB due to the activation of certain factors, resulting in the activation of NF-κB, which in turn leads to a large number of inflammatory factor gene expressions [19]. The experimental results show that GAA significantly inhibited TLR4 pathway.

In conclusion, this study indicates that GAA reduced inflammatory reaction through TLR/NF-κB signaling pathway and played an anti-asthma role in asthmatic mice.
